# Identification of shared and unique mechanisms of atopic dermatitis and ulcerative colitis by construction and computational analysis of disease maps

**DOI:** 10.1016/j.csbj.2025.09.008

**Published:** 2025-09-07

**Authors:** Oxana Lopata, Marcio Luis Acencio, Xinhui Wang, Ahmed Abdelmonem Hemedan, Michael J. Chao, Scott A. Jelinsky, Florian Tran, Philip Rosenstiel, Andrew Y.F. Li Yim, Reinhard Schneider, Venkata Satagopam, Marek Ostaszewski

**Affiliations:** a Luxembourg Centre for Systems Biomedicine, University of Luxembourg, Esch-sur-Alzette, Luxembourg; b ELIXIR Luxembourg, Esch-sur-Alzette, Luxembourg; c Sanofi, Cambridge, MA, USA; d Department of Internal Medicine I, University Hospital Schleswig-Holstein, Campus Kiel, Kiel, Germany; e Institute of Clinical Molecular Biology, Kiel University, Kiel, Germany; f Pfizer Inc, Cambridge, MA, USA; g Tytgat Institute for Liver and Intestinal Research, Amsterdam University Medical Centers, University of Amsterdam, the Netherlands

**Keywords:** Systems biology, Systems biomedicine, Ulcerative colitis, Atopic dermatitis, Biomarkers, Disease maps

## Abstract

Atopic dermatitis (AD) and ulcerative colitis (UC) are immune-mediated inflammatory diseases (IMIDs) with high prevalence and treatment costs. AD mainly affects the skin, while UC targets the colon and rectum, but both are characterised by immune dysregulation driven by aberrant T helper cell activation, persistent barrier dysfunction, genetic predisposition, and environmental triggers. This overlap may explain the link between the two diseases and the increased risk of UC in patients with AD. Both diseases are chronic, progressive, and limited in treatment options, and there is a need for a better understanding of their mechanisms and biomarkers. To address this, we developed disease maps for UC and AD, covering their molecular mechanisms. Here, we present the development and contents of the maps, as well as demonstrate their application in data visualisation and analysis. Our systematic, interactive comparison reveals both common and disease-specific signatures, as well as common pathological pathways. These findings highlight shared biomarkers for predicting progression and therapy outcomes, and opportunities for drug repurposing. The UC and AD disease maps provide a valuable resource for representing and exploring common and distinct mechanisms, helping to advance IMID management from organ-based symptom relief towards mechanism-based treatments.

## Graphical Abstract

**Figure:**
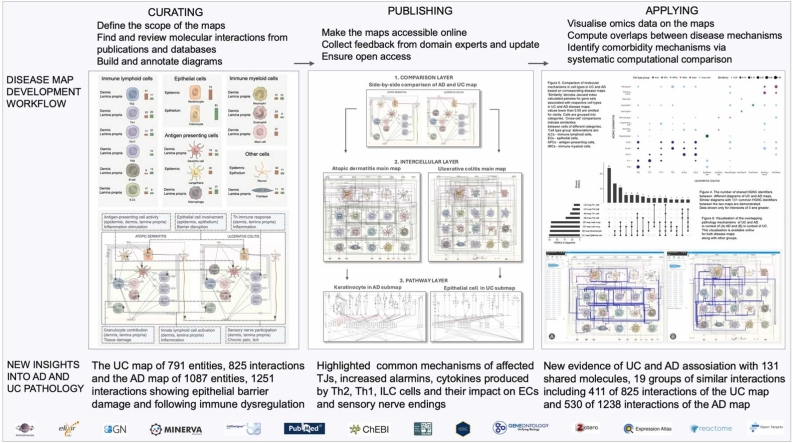

## 1 Introduction

Immune-mediated inflammatory diseases (IMIDs) are disorders caused by immune system dysregulation that leads to persistent inflammation, tissue damage, physical pain, limited functional abilities, a reduced quality of life, and, in some cases, premature death [1], [2]. Many patients are either unresponsive to treatment or relapse after initially successful therapy, with a risk of developing additional IMIDs [3].

Diseases such as inflammatory arthritis, inflammatory bowel disease (IBD), and inflammatory skin disorders manifest across different organs and tissues. There is increasing evidence pointing to a connection between chronic skin diseases and gastrointestinal disorders [4], [5]. Ulcerative colitis (UC) and atopic dermatitis (AD) are two IMIDs with shared etiological factors.

UC is a chronic IBD of the colon that causes recurring inflammation and ulcers in the mucosal lining, typically beginning in the rectum and extending upward [6]. AD is a recurrent, chronic inflammatory skin disease characterised by impaired epidermal barrier function, severe skin inflammation, frequent cutaneous infections, and pruritus [7]. Both conditions involve barrier dysfunction that contributes to their pathogenesis: UC in the intestinal lining and AD in the skin [8], [9].

AD is associated with multiple comorbidities, including UC [4], [10]. A higher risk of UC in AD patients has been shown in retrospective cohort studies from the UK and Germany [11], [12], in Japanese [13] and Korean [14] populations, and through meta-analysis when examining cross-disease associations [5]. Genetic studies further support this connection, with Mendelian randomisation (MR) analysis in European [15] and East Asian [16] AD populations pointing to causal relationships between the two diseases.

These connections suggest shared pathological molecular pathways that contribute to the development of UC and AD. However current resources lack the capacity for studying such pathways in these complex conditions. To address this, we present the UC and AD disease maps - computational and diagrammatic models of molecular mechanisms implicated in both disorders, developed according to systems biology standards and based on curated biomedical research data.

The maps were created through systematic literature curation, constructed using established systems biomedicine methods [17], [18], and refined with omics databases to ensure their relevance. They provide an interactive graphical tool for integrating disease mechanisms and reviewing pathological pathways. As a standardised knowledge resource, the maps consist of interconnected diagrams built according to community guidelines [19], and workflows (https://disease-maps.io). The maps are openly accessible online individually at https://imi-immuniverse.elixir-luxembourg.org/minerva/?id=UCmaps31-01–25 and https://imi-immuniverse.elixir-luxembourg.org/minerva/id=ADmaps31-01–25, and as an overview,^1^ supporting interactive exploration as well as computational workflows, network analysis and modelling approaches.

In this article, we outline the design of the UC and AD disease maps, including their hierarchy and connectivity. We describe the methods used for development, encoding interactions into diagrams, and annotating map elements. We analyse disease phenotypes and identify common and disease-specific mechanisms. We further demonstrate the maps’ application by integrating UC and AD genetic risk factors from the OpenTargets platform and expression profiles from ExpressionAtlas. Finally, we programmatically compare UC and AD disease maps to identify shared molecules, cell types, molecular network modules and potential drug targets.

## 2 Results

The UC map is a state-of-the-art resource incorporating data from 325 disease-relevant publications with 791 entities, 825 interactions, and 327 unique molecular entities (proteins, RNAs, genes, and simple molecules). The AD map is developed using 478 publications with 1087 entities, 1251 interactions, and 391 unique molecular entities. Each entity is confirmed with a reference to a publication with experimentally validated evidence.

Both maps are openly accessible as interactive online resources available at https://imi-immuniverse.elixir-luxembourg.org/minerva/?id=UCmaps31-01–25 and https://imi-immuniverse.elixir-luxembourg.org/minerva/id=ADmaps31-01–25, with user guides and licensing information provided at https://disease-maps.io/ucadmap.

In the following sections, we describe their design, detail the contents of their diagrams, and present use cases for their application.

### 2.1 Structure of UC and AD maps

The maps represent knowledge from publications on UC and AD molecular and cellular mechanisms. Their contents were developed through manual curation of scientific literature guided by focused searches and clinical expertise and refined with computational analysis. This information was encoded at the level of intra- and intercellular interactions in a comprehensive, systematic manner, following an established protocol.

**Fig. 1:**
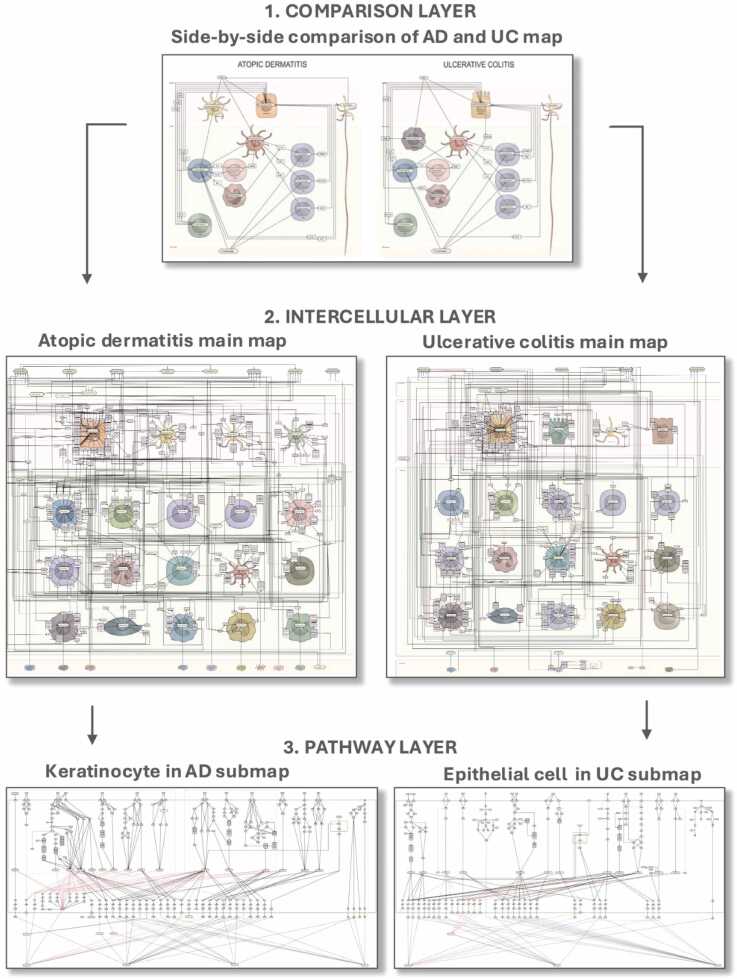
A schematic review of the UC and AD disease maps. The first layer is a compact illustrative side-by-side comparison of UC and AD mechanisms. The second layer shows individual maps of UC and AD with cells and interactions between them through cytokines, chemokines, simple molecules and corresponding receptors. The third layer describes specific pathways in key cell types for each disease. The keratinocyte diagram for AD and the epithelial cell diagram for UC are shown as an example. The user guide is available online at https://disease-maps.io/ucadmap.

The UC and AD disease maps are organised into three layers (Fig. 1):

1. Comparison layer - a compact, illustrative side-by-side overview of key cell types, cytokines, and signalling pathways. This simplified representation serves as an entry point to the detailed UC and AD maps.
2. Intercellular layer - a network of key cell types and molecular interactions, including signalling processes represented in diagrams.
3. Pathway layer - disease and cell type-specific pathways composed of metabolic, signalling, and gene regulatory interactions.

The intercellular and pathway layers follow systems biology standards and are both human- and computer-readable.

### 2.2 Comparison layer: side-by-side representation of key UC and AD mechanisms

**Fig. 2:**
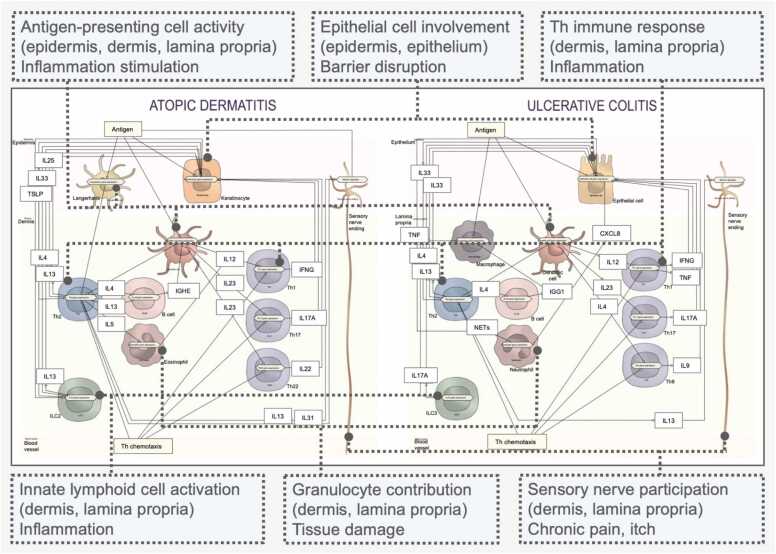
A side-by-side comparison of UC and AD, representing shared mechanisms. Similar cell types and their involvement in disease processes are shown with boxes and connectors.

UC and AD are distinct conditions with unique characteristics and manifestations, but they also share underlying mechanisms. Although the UC and AD pathology maps were constructed independently, certain mechanism-related features are relevant for both diseases. These are summarised in the disease comparison layer (Fig. 2).

The comparison map shows the following common mechanisms:

− epithelial cells (keratinocytes, colonocytes): barrier disruption and initiation of inflammation;
− antigen-presenting cells (Langerhans cells, dendritic cells, macrophages): activation of T cell differentiation and B cell stimulation with increased Ig levels;
− T helper cell responses (Th2, Th1, Th17, Th22, Th9): cytokine production that disturbs epithelial cell proliferation/differentiation and drives inflammation;
− innate lymphoid cells (ILC2, ILC3): contribution to inflammatory responses;
− granulocytes (eosinophils, neutrophils): tissue damage during pathogenesis;
− sensory nerves: activation leading to chronic pain and itching.

Detailed descriptions of the molecular mechanisms behind these phenotypes are provided in Supplementary Material S1.

### 2.3 Intercellular layer: UC and AD maps

**The intercellular UC map**^2^ (Intercellular layer in Fig. 1, Supplementary Material S2) is a molecular network featuring 19 cell types across three tissues. In the colonic epithelium layer, four key cell types most affected by pathology are epithelial cells, goblet cells, primary afferent neurons, and enteroendocrine cells. The lamina propria layer beneath the epithelium includes Th naïve cells, Th2, Th1, Th9, Th17, Th22, dendritic cells, ILC3s, macrophages, eosinophils, B cells, neutrophils, and their mediator production. The blood vessel layer shows chemotaxis of innate and adaptive immune cells into the lamina propria. UC pathology is linked to three major factors: (i) impaired epithelial barrier function through disruption of colonic epithelial tight junctions, (ii) dysregulated immune responses, and (iii) visceral pain. The intercellular UC map illustrates how epithelial barrier disruption drives immune dysregulation and associated pathological mechanisms via cytokines, chemokines, and other mediators. A detailed list of molecular events, with links to their location in the diagram, is provided in Supplementary Material S2.

**The intercellular AD map**^3^ (Intercellular layer in Fig. 1, Supplementary Material S3) also represents a molecular network of 19 cell types across three tissues. In the epidermis layer, the main cell types involved in AD pathology are keratinocytes, Langerhans cells, inflammatory dendritic epidermal cells, and pruriceptive primary afferent neurons. The dermis layer includes Th naïve cells and their differentiation into Th2, Th1, Th17, and Th22, along with dermal dendritic cells, ILC2s, mast cells, eosinophils, B cells, neutrophils, and their cytokine milieu. The third layer shows chemotaxis of innate and adaptive immune cells from peripheral blood into the skin. This network demonstrates how skin barrier dysfunction triggers a cascade of Th-mediated inflammation, with pruritus further amplifying the cycle, visualised through cell-to-cell communication in the intercellular layer. A detailed list of molecular events, with links to their location in the diagram, is provided in Supplementary Material S3.

### 2.4 Pathway layer: shared pathology mechanisms

Various IMIDs share inflammatory pathways linked to dysregulation of the innate and adaptive immune systems. In the UC and AD disease maps, these mechanisms are detailed in the pathway layer (Fig. 1, Supplementary Material S4). Key pathways include NFκB, JAK-STAT, and MEK-ERK signalling in colonocytes (UC) and keratinocytes (AD), activated by immune-cell-derived IL-13, IL-17A, IL-22, and IFNG. These signals lead to downregulation of tight junction proteins and upregulation of alarmins, S100 proteins, and IL-8. Additional pathways involve IRAK–TRAF6–MAPK signalling activated by IL-33, and JAK-STAT6–GATA3 signalling activated by IL-4 in Th2 cells, which upregulates Th2 cytokines such as IL-13 and IL-5. Finally, IRAK and NFκB signalling in Th1 cells, initiated by IL-18 from epithelial and dendritic cells, together with a JAK-STAT1-TBX21 autocrine loop, promote IFNG production. A full list of mechanisms and cell type–specific submaps is provided in Supplementary Material S4.

**Fig. 3:**
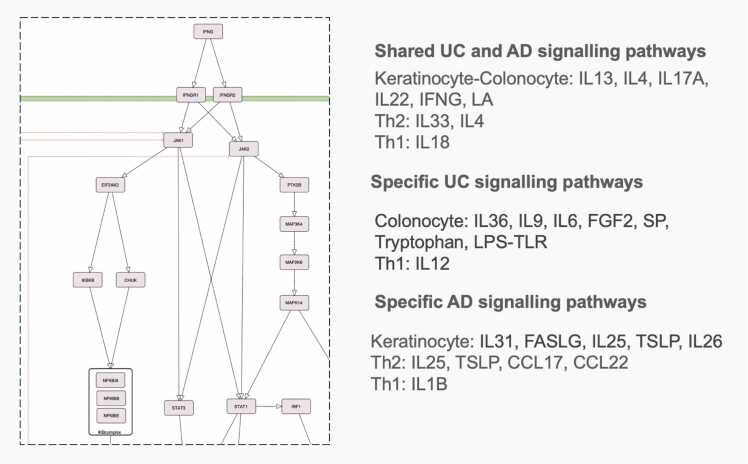
Intracellular mechanisms in the pathway layer. Common and unique signalling pathways in submaps of key cell types for UC and AD are listed.

### 2.5 Exploration of the UC and AD maps

UC and AD maps are computational resources, supporting reproducible visualisation and analysis of omics data in the context of disease. Here we introduce use cases of such applications, which can be reused as new data or map content appears.

#### 2.5.1 Visual exploration of omics data

To illustrate how gene variants influence downstream molecular events represented in the maps, we collected UC-associated variants from the Open Targets Genetics database [20], focusing on single nucleotide polymorphisms (SNPs). The data was formatted as an overlay on the UC map, highlighting overlaps with disease mechanisms and pinpointing the most relevant pathways and cell types. A similar dataset was generated for the AD map.

**Fig. 4:**
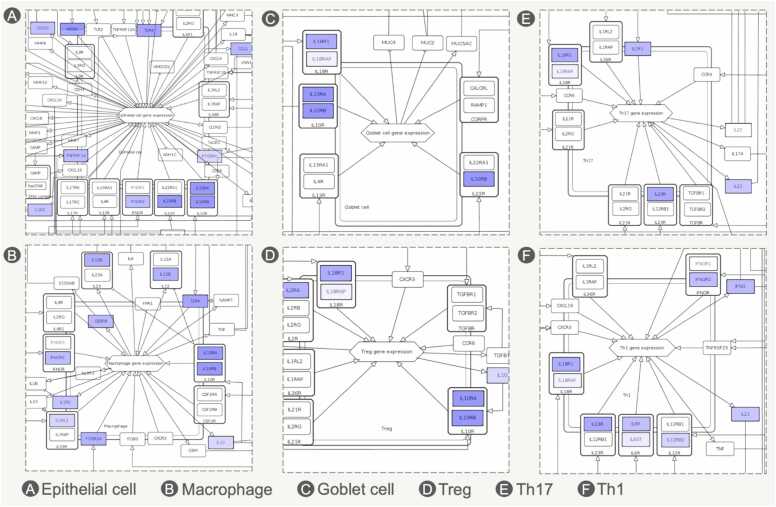
Visualisation of UC-associated gene variants across different cell types. UC-linked SNPs, highlighted in blue, are represented in immune and epithelial cell types in the UC disease map, based on Open Targets Genetics data.

Additionally, we retrieved data from ExpressionAtlas (https://www.ebi.ac.uk/gxa/) using the keywords “colitis” and “dermatitis” in humans. The results were normalised and visualised in both maps to provide further insight into affected molecular mechanisms.

These genetic and expression overlays are openly accessible in the UC and AD maps, with a user guide available at https://disease-maps.io/ucadmap.

#### 2.5.2 Programmatic comparison of the UC and AD maps contents

**Fig. 5:**
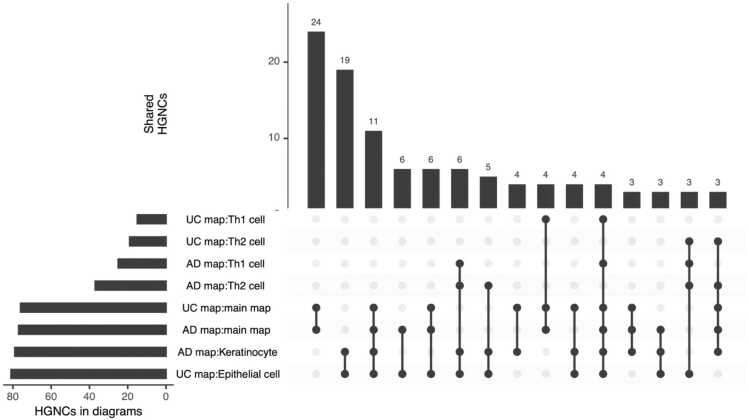
The number of shared HGNC identifiers between different diagrams of UC and AD maps. Similar diagrams with 131 common HGNC identifiers are demonstrated (only overlaps of three or more).

To identify common and disease-specific mechanisms across UC and AD, we performed a systematic computational analysis of their contents following Gawron et al. [21]. First we compared stable HGNC identifiers (HUGO Gene Nomenclature Committee, www.genenames.org) shared between the maps. The UC map contains 230 unique identifiers and the AD map - 270, with 131 in common. Fig. 5 shows the most similar diagrams, and Table 1 highlights the ten most frequently shared HGNC symbols and the diagrams in which they appear. Similarly, disease-specific coverage can be indicated (see Supplementary Material S5).

**Table 1:** Gene symbols of the top ten molecules common in UC and AD disease maps, indicating in which diagrams they appear.

	**AD map**				**UC map**			
**HGNC symbol**	**Main map**	**Keratinocyte**	**Th1** **cell**	**Th2** **cell**	**Main map**	**Epithelial cell**	**Th1** **cell**	**Th2** **cell**
IL1RAP	v	v	v	v	v	v		v
IFNG	v	v	v		v	v	v	
IFNGR1	v	v	v		v	v	v	
IFNGR2	v	v	v		v	v	v	
IL13	v	v		v	v	v		v
IL18	v	v	v		v	v	v	
IL4R	v	v		v	v	v		v
JAK1		v	v	v		v	v	v
IL17RA	v	v		v	v	v		
IL18R1	v	v	v		v		v	

**Fig. 6:**
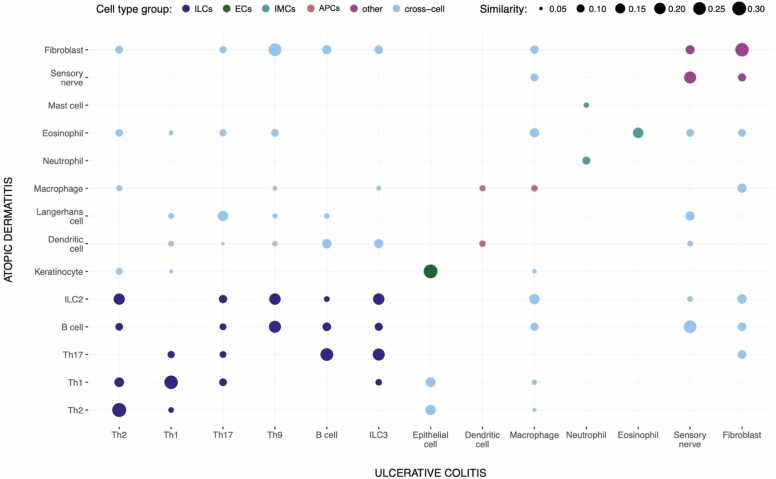
Cell type similarity between UC and AD based on Jaccard index values for gene sets (values ≤0.05 omitted). Cell types are grouped into categories (ILCs - immune lymphoid cells, ECs - epithelial cells, APCs - antigen-presenting cells, IMCs - immune myeloid cells). Cross-cell comparisons indicate similarities across categories.

To assess cell-type similarities, we retrieved lists of proteins for each cell type and calculated Jaccard index values using HGNC symbols. Results are shown in Fig. 6, with cell types grouped for clarity and values ≤ 0.05 omitted. A high similarity was observed among immune lymphoid cells, while antigen-presenting cells showed a comparatively lower overlap. Gut epithelial cells in UC and keratinocytes in AD showed similar molecular signatures, overlapping with Th2 and Th1 profiles. Supplementary Material S6 illustrates which cell-related genes in each map are disease specific.

**Fig. 7:**
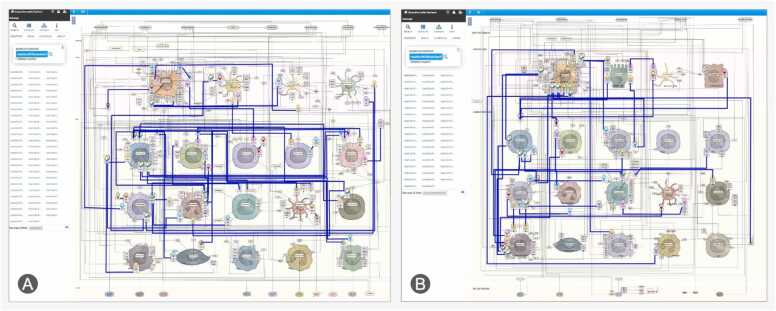
Visualisation of the overlapping pathology mechanisms, shown in context of (A) AD and (B) UC. This visualisation is available online for both disease maps along with other groups (see Supplementary Material S7). Specific connections can be accessed from the table using the URLs provided.

To compare the structure of both maps, we calculated a score for overlapping interactions, grouped them and measured their overlap. This revealed 19 areas of significant similarity. Fig. 7 illustrates two examples, high-resolution interactive versions are available online (Supplementary Material S7).

**Fig. 8:**
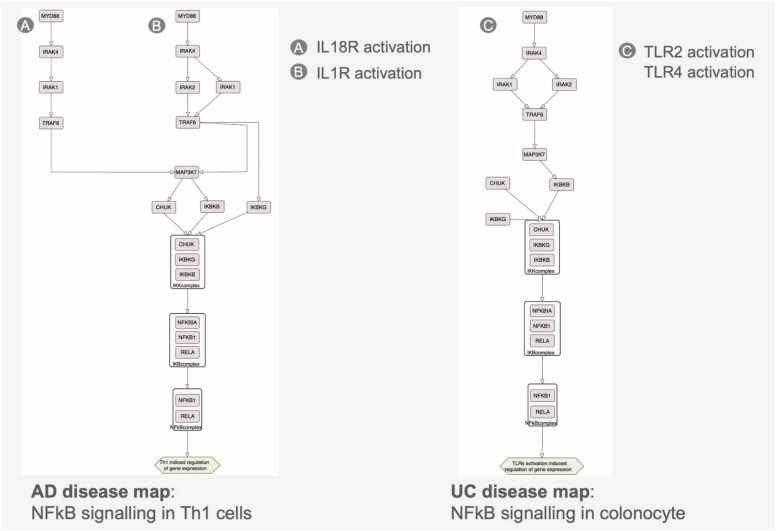
Comparison of NFkB signalling in AD and UC maps across different cell types, showing different regulation of similar core mechanisms.

A detailed example is shown in Fig. 8, comparing NFκB signalling in Th1 cells in AD to epithelial cells in UC. Both maps reveal overlapping inflammatory pathways, including IL-13, IL-17, IL-22, and IFNG effects on epithelial cells, Th activation by IL-6, eosinophil activation by IL-5, and Th1 differentiation induced by IL-12.

Overall, 19 groups of similar interactions include 411 of 825 UC interactions, and 530 of 1238 AD interactions, with TRAF6, MAP3K7, CHUK, IKBKB and IKBKG present across 7 or more groups. Please see Supplementary Material S8 for a complete list of similar groups, their content and direct links in corresponding disease maps. To complete this picture and highlight differences, we calculated map clusters which are disease specific. It shows reactions dissimilar to any reaction in the other map (Supplementary Materials S9a and S9b).

Next, we focused on overlapping proteins that are known drug targets. Using DrugBank [22] and ChEMBL [23], we identified targets for UC and AD molecular network overlays. Supplementary Material S10 lists all drugs that act on elements within the 19 common groups of interactions. These drugs are in clinical use or trials, with approval and application supported by literature references. We found 28 shared targets, primarily proinflammatory cytokines, their receptors, and chemotaxis-related molecules, with 171 drugs acting on these targets.

We then grouped drugs by the 19 interaction clusters, considering only those with more than one target per group (Supplementary Material S11). The largest numbers were linked to NFκB, MAPK, and JAK signalling pathways. A literature review of all 28 drug targets confirmed JAK-STAT signalling as a key therapeutic focus. Activation of this pathway by cytokines in UC and AD drives Th2 and Th1 responses, eosinophil activation, and epithelial cell damage. Notably, the JAK inhibitors baricitinib and abrocitinib, approved for AD, show promising potential for repurposing in UC, supported by emerging preclinical evidence [24], [25], [26], [27].

Overall, our drug analysis demonstrates that integrating UC and AD disease maps enables the identification of shared molecular targets and presents opportunities for cross-disease therapy.

## 3 Discussion

A systematic review of the literature highlights the complexity of UC and AD mechanisms. Disease maps integrate this knowledge into structured computational resources, enabling systematic comparison, hypothesis generation and identification of therapeutic strategies.

### 3.1 Shared UC and AD mechanisms

Both disorders are inflammatory diseases of epithelial surfaces: UC affects colonic epithelium, while AD affects the skin epithelium. Pathology alters epithelial structure and function, leading to hyperplasia or barrier dysfunction - epithelial abnormalities in UC [28] and an impaired skin barrier in AD [29]. Colonocytes and keratinocytes are influenced by cytokines such as IL-13, IL-4, IL-17, IL-22, and IFNG, which stimulate alarmin expression (e.g., IL-33) [30], [31]. Additional cytokines are disease-specific: IL-25 and TSLP in AD [32], [33], and IL-8 in UC [34]. Both maps illustrate these mechanisms [35] and show IL-13 as a central mediator of barrier loss. In AD, IL-13 from Th2, ILCs, mast cells, and NKT cells acts on keratinocytes via mTOR-AKT signalling, downregulating FLG [36], Loricrin, Involucrin, and FLG2, proteins essential for epidermal integrity [37], [38]. In UC, IL-13 downregulates tight junction proteins Claudin-8, Occludin [39], and tricellulin [40], while upregulating Claudin-2 [41], a pore-forming protein that increases epithelial permeability.

T helper cell dysregulation is another shared feature. Acute AD is dominated by Th2 cytokines (IL-4, IL-13, IL-5) [42], while chronic AD involves Th1, Th17, and Th22 subsets [43]. Certain endotypes (Asian, paediatric, intrinsic) show strong Th17/IL-17 activity [35]. UC predominantly involves Th1 (IFNG, TNF) and Th17 (IL-17) responses that drive gut inflammation [44]. Some UC endotypes activate Th2 cytokines (IL-4, IL-13, IL-5) [41], and Th9 also contributes to epithelial pathology [45]. Both maps capture how IL-4 signalling in Th2 cells enhances inflammation via JAK-STAT6 [46], GATA3 [47] in UC and AD [48], [49]. IL-18 further increases Th1 responses in both diseases: derived from keratinocytes [50] and inflammatory dendritic epidermal cells [51] in AD to enhanced IFNG production through IRAK and NFkB [52], [53], and from colonocytes [54] or dendritic cells [55] in UC, with increased IFNG production [56]. IFNG itself increases Th1 activation via an autocrine JAK-STAT1-TBX21 loop in AD [57] and UC [58], [59].

Innate lymphoid cells (ILCs) also contribute: ILC2 in AD produces IL-4, IL-13, and IL-5, driving chronic inflammation [60], while ILC3 in UC produces IL-17, promoting gut pathology [61].Granulocytes are similarly involved: eosinophils in AD increase inflammation by releasing IL-12 [62], whereas neutrophils in UC form extracellular traps that injure tissue and promote inflammation [63].

Sensory nerve activation links both diseases to chronic pain. In AD, mediators such as IL-31 and IL-13 sensitise cutaneous sensory nerves, intensifying itching and pain [64]. In UC, gut inflammation activates visceral sensory neurons, with IL-13 from Th2 and NKT cells playing a role in the process [65].

Detailed representations of these intercellular and pathway-level mechanisms are available in Supplementary Materials S2, S3, and S4.

### 3.2 Computational analysis

Analysis of UC and AD disease maps for overlap (Fig. 6, Table 1) reveals shared biomarkers and common pathological pathways. Table 1 lists the most common proteins in both diseases, reflecting an inflammation-related signature. They include Th2 and Th1 cytokines and their receptors - IL-13, IL-4R, IFNG, and IFNGR1/IFNGR2 - reinforcing the role of Th2 and Th1 responses in UC and AD pathogenesis. IL-13 and IL-4 are potential plasma biomarkers in AD [60], while serum IL-13 is a candidate biomarker in UC [61]. IL-18 promotes Th0 differentiation into Th1 cells and stimulates IFNG production, contributing to pathology in both AD [62] and UC [63], [64].

Fig. 6 illustrates cell-type similarities between UC and AD. Immune lymphoid cells show high similarity for Th2, Th1, B cells and ILCs. In cross-category comparisons, ILC2 in AD [60], [66], [67]and Th2 in UC [41] share similar proinflammatory cytokine profiles: IL-4, IL-13, and IL-5. In both diseases these molecules drive epithelial damage and eosinophil activation. [41], [60], [68], [69]. Another example is Th17 in AD and ILC3 in UC**.** Their IL-17 release contributes to intestinal inflammation in UC [61], and in certain AD endotypes [35] to tissue damage and chronic skin inflammation in AD [70]. Immune myeloid cells (eosinophils, neutrophils), fibroblasts, and sensory nerve endings show similar molecular activity, supporting mechanisms of tissue injury, chronic inflammation, and pain [62], [63], [64], [65].

Computational analysis also identified specific network areas in maps with overlapping molecular mechanisms. Fig. 8 shows one of the 19 groups, highlighting NFκB signalling in UC and AD across different cell types. Despite various upstream triggers, similar downstream events occur: IL-1R activation in Th1 cells in AD and TLR activation in epithelial cells in UC converge on TRAF6, IKBKG, and MAP3K7, responding to IL-1R ligands (IL-18, IL-1B) and TLR ligands (LPS) [71], [72], [73].

Therapeutic development for UC and AD remains limited, although JAK inhibitors have shown efficacy. Tofacitinib [74] and upadacitinib [75] are effective in patients with both UC and AD, targeting the JAK-STAT pathway, a key driver of inflammation in both conditions. Our evaluation highlights JAK-STAT signalling as an active area for drug discovery and repurposing. For example, baricitinib, a selective JAK1/2 inhibitor approved for AD [24], [25], has demonstrated safety and efficacy in clinical trials and shows promise in preclinical models of UC [76]. Similarly, abrocitinib, a JAK1 inhibitor recently approved in Europe for AD [26], [77], might have therapeutic relevance for UC based on shared mechanisms, though it is not yet in clinical trials for it [27].

Overall, computational analysis of the UC and AD maps identifies disease-specific and shared biomarkers, clarifying overlapping mechanisms across cell types and highlighting potential treatment targets. These workflows are reproducible and can generate new insights as the maps are further developed by the research community. This approach allows for investigations into UC and AD comorbidities, supports data interpretation and advances research toward mechanism-based therapeutic strategies. Similar analyses can be performed for other diseases using additional maps and pathway databases.

### 3.3 Perspectives and future updates

The UC and AD disease maps were primarily developed through a systematic curation of PubMed articles to capture current knowledge of disease mechanisms. As new research is published, the maps will be regularly updated to maintain relevance. Such updates are facilitated by the resource structure, and the effort is supported by the ImmUniverse consortium (https://www.immuniverse.eu/).

The maps’ content has been evaluated by domain experts and expanded based on feedback from the consortium. While it is challenging to capture all published information, the systematic methodology [19] ensures coverage of major disease areas and their underlying mechanisms.

## 4 Materials and methods

### 4.1 Map construction

Molecular mechanisms of UC and AD were extracted from biomedical literature and encoded into disease maps using CellDesigner (https://www.celldesigner.org), a diagram editor that follows the Systems Biology Graphical Notation (SBGN) standard [78]. It is connected to the MINERVA (Molecular Interaction NEtwoRks VisuAlisation) platform [79] for interactive visualisation and exploration.

#### 4.1.1 Literature search

Our curation process follows the community guidelines [19]. We collected evidence from biomedical publications, confirming the relevance of molecular interactions for UC or AD. Each interaction is supported by experimentally validated data from disease-relevant papers and pathway databases, including PubMed, PubMed Central, Reactome [80], KEGG [81], UniProt (https://www.uniprot.org), and CHEBI (https://www.ebi.ac.uk/chebi). References for each interaction are provided as PMID or DOI.

A total of 803 papers were selected: 325 for UC and 478 for AD intercellular maps and submaps. First, key papers suggested by domain experts from the ImmUniverse consortium were reviewed. Afterwards a broader search incorporated general experimental data for immune-inflammatory conditions to fill in the gaps in disease pathology. Pathway databases were also used when necessary to complete downstream events. Maps were updated according to the feedback from domain experts, including the integration of genes linked to UC and AD. Every step followed a protocol ensuring that each gene or protein is experimentally validated in the relevant cell type and tissue.

#### 4.1.2 Encoding interactions into diagrams

Molecular interactions from publications were manually integrated, reviewed, and represented as diagrams [82]. CellDesigner was used to create, edit, and update UC and AD disease maps [83]. The editor supports extensive diagrams in Process Description and Activity Flow formats and enables SBGN-compatible representations. We used SBGN [78] ActivityFlow to depict molecules and their communications.

#### 4.1.3 Annotation of map elements and interactions

Each entity in the maps is identified and linked to the corresponding external database. Proteins, RNAs, and genes are named according to HGNC standards and annotated with UniProt IDs. Simple molecules are annotated via CHEBI, while phenotypes are annotated using Gene Ontology (GO) biological process terms or MeSH disease-related terms.

Automatic annotation in MINERVA is possible for entities with HGNC names. Manual annotation is required when generic names or synonyms prevent automatic recognition, particularly for metabolic entities and phenotypes. All interactions are annotated with PMIDs or DOIs. Disease-specific evidence is extracted from the literature, and all articles used in this project are stored in a Zotero library.

### 4.2 Maps availability and visualisation in the MINERVA platform

The MINERVA platform [79] provides a web-based interface for exploring UC and AD disease maps and representing molecular interaction networks, available at https://imi-immuniverse.elixir-luxembourg.org/minerva/index.html?id=UCmaps31-01–25 and https://imi-immuniverse.elixir-luxembourg.org/minerva/index.html?id=ADmaps31-01–25 respectively. Users can browse the maps, view descriptions, search for elements (molecules, interactions, publications), and overlay datasets such as transcriptomics or gene variant data, which are visualised as color-coded representations on the maps.

### 4.3 Construction of data overlays

Data overlays for UC and AD maps were generated using Open Targets [84] and Expression Atlas [85]. On 27th September 2024, Open Targets was queried for genetic variants associated with UC (EFO_0000729) and AD (EFO_0000274), producing lists of genes affected by at least one variant.

Expression Atlas was queried on 28th May 2024 using the terms “colitis” and “dermatitis” for humans. Log fold change values from both datasets were scaled to the [-1, 1] range for visualisation in MINERVA.

UC and AD maps were compared according to the workflow in the publication by Gawron, P. et al. [21], with HGNC symbols serving as element identifiers. Pairwise similarity between map interactions was calculated using the metric in the original workflow, keeping only interactions with similarity ≥ 0.7 and at least two matching elements. Strongly connected components (SCCs) were identified for these interactions and compared pairwise between maps. SCC pairs with five or more similar reactions were considered groups of similar interactions.

### 4.4 Drug target analysis

Drug targets were retrieved using the minervar package v0.9.1 [21] (https://gitlab.com/uniluxembourg/lcsb/BioCore/minerva/minervar) for MINERVA-hosted UC and AD maps. Entries from DrugBank and ChEMBL were collected for elements within the 19 groups of similar interactions identified in Section 4.4.

## 5 Conclusion

UC and AD are known to be related, AD patients exhibiting an increased risk of developing UC. This study focuses on the shared and unique molecular mechanisms of both diseases, constructing UC and AD disease maps, followed by computational analysis. Despite different clinical manifestations, both conditions exhibit an epithelial barrier dysfunction, immune dysregulation (including Th2, Th1, and ILC responses) and sensory nerve activation. Key inflammatory pathways identified in both diseases include NFκB, MEK-ERK, IRAK-TRAF6-MAPK, JAK-STAT6-GATA3, and JAK-STAT1-TBX21, showing overlapping mechanisms of chronic inflammation and tissue damage. Computational analysis also revealed shared biomarkers such as IL-13, IL-4R, IFNG, and IL-18, showing potential for drug repurposing.

The disease maps are community-constructed knowledge repositories, translating visually legible, pathology-specific pathway diagrams into computational resources for analysis and modelling. These workflows are reusable, allowing for integration of new data as the maps are updated. By providing an interactive, open-access platform, the UC and AD disease maps support future research and predictive modelling for IMIDs. Continuous updates will enhance understanding of disease mechanisms and help the development of novel treatments to improve patient outcomes.

## Funding source

This project has received funding from the Innovative Medicines Initiative 2 Joint Undertaking (JU) under grant agreement No. 853995 (ImmUniverse). The JU receives support from the European Union’s Horizon 2020 research and innovation programme and 10.13039/100013322EFPIA.

## CRediT authorship contribution statement

**Scott A. Jelinsky:** Writing – review & editing, Writing – original draft, Validation, Formal analysis. **Florian Tran:** Writing – review & editing, Writing – original draft, Validation, Formal analysis. **Michael J. Chao:** Writing – review & editing, Validation, Supervision. **Reinhard Schneider:** Writing – review & editing, Supervision, Resources, Conceptualization. **Venkata Satagopam:** Writing – review & editing, Supervision, Resources, Conceptualization. **Philip Rosenstiel:** Writing – review & editing, Validation, Formal analysis. **Andrew Y.F. Li Yim:** Writing – review & editing, Writing – original draft, Validation. **Xinhui Wang:** Writing – review & editing, Project administration, Conceptualization. **Ahmed Abdelmonem Hemedan:** Writing – review & editing, Visualization, Methodology, Data curation, Conceptualization. **Marek Ostaszewski:** Writing – review & editing, Writing – original draft, Visualization, Supervision, Resources, Methodology, Investigation, Data curation, Conceptualization. **Oxana Lopata:** Writing – review & editing, Writing – original draft, Visualization, Methodology, Investigation, Formal analysis, Data curation, Conceptualization. **Marcio Luis Acencio:** Writing – review & editing, Methodology, Data curation, Conceptualization.

## Declaration of Competing Interest

Andrew Y.F. Li Yim receives grants from Amsterdam UMC and Crohn’s and Colitis Foundation, consulting fees from DeciBio, payment from Janssen and Johnson&Johnson, and has GSK stocks.

## Data Availability

The data underlying this article are available in the article, in the supplementary materials, and the maps are freely available via the MINERVA platform. The data supporting the conclusions of this article are included within the article.

## Footnotes

Appendix A Supplementary data associated with this article can be found in the online version at doi:10.1016/j.csbj.2025.09.008.

## Footnotes

1 https://imi-immuniverse.elixir-luxembourg.org

2 https://imi-immuniverse.elixir-luxembourg.org/minerva/?id=UCmaps31–01–25

3 https://imi-immuniverse.elixir-luxembourg.org/minerva/?id=ADmaps31–01–25
