# Supplementary material for: Identification of shared and unique mechanisms of atopic dermatitis and ulcerative colitis by construction and computational analysis of disease maps
Source: Comput Struct Biotechnol J. 2025 Sep 7;27:4007–18. doi: 10.1016/j.csbj.2025.09.008 (PMC12465054; doi:10.1016/j.csbj.2025.09.008)
Supplement: Supplementary file 2 — Supplementary material [file mmc2.docx]

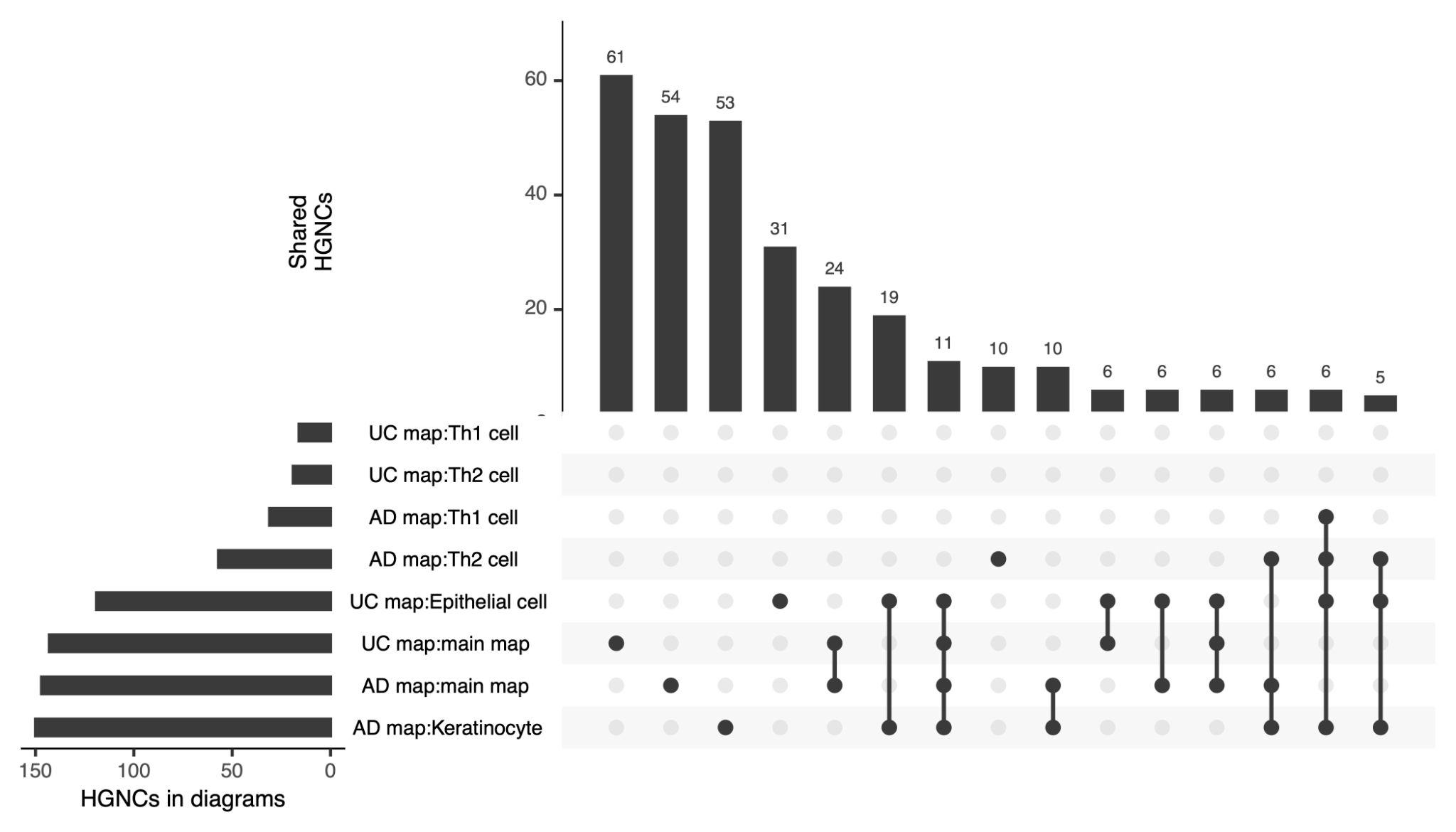


**Figure A**. The number of HGNC identifiers in diagrams of UC and AD maps, including 500 disease specific (230 unique for UC and 270 unique for AD) and 131 shared for both maps.
