# Supplementary material for: Identification of shared and unique mechanisms of atopic dermatitis and ulcerative colitis by construction and computational analysis of disease maps
Source: Comput Struct Biotechnol J. 2025 Sep 7;27:4007–18. doi: 10.1016/j.csbj.2025.09.008 (PMC12465054; doi:10.1016/j.csbj.2025.09.008)
Supplement: Supplementary file 3 — Supplementary material [file mmc3.docx]

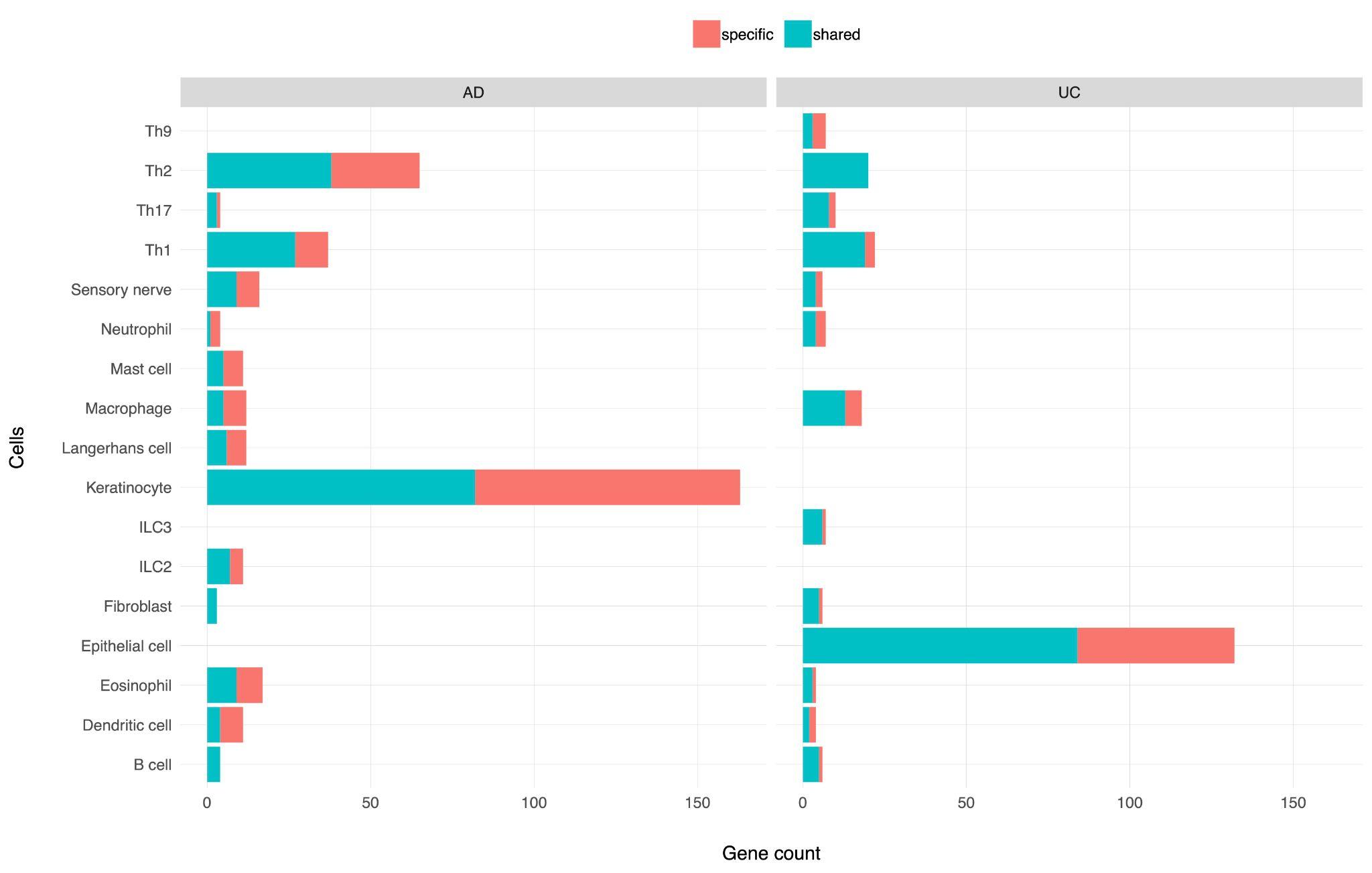


**Figure B**. Cell-related gene comparison between UC and AD. Disease-specific genes for each cell type are highlighted in red, while genes common to both UC and AD are shown in green.

This visualisation helps to distinguish unique molecular features of each disease and also emphasises the overlapping parts.
