# Supplementary figures and images for: Identification of shared and unique mechanisms of atopic dermatitis and ulcerative colitis by construction and computational analysis of disease maps
Source: Comput Struct Biotechnol J. 2025 Sep 7;27:4007–18. doi: 10.1016/j.csbj.2025.09.008 (PMC12465054; doi:10.1016/j.csbj.2025.09.008)

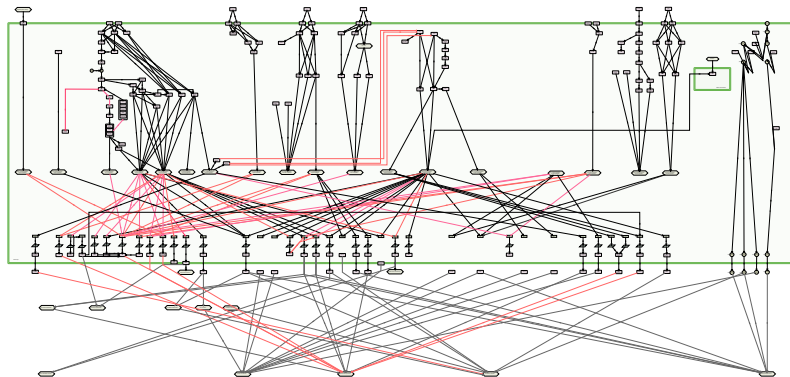

Supplement: Supplementary file 7 — Supplementary material [file mmc7.zip › AD_218.xml.pdf]

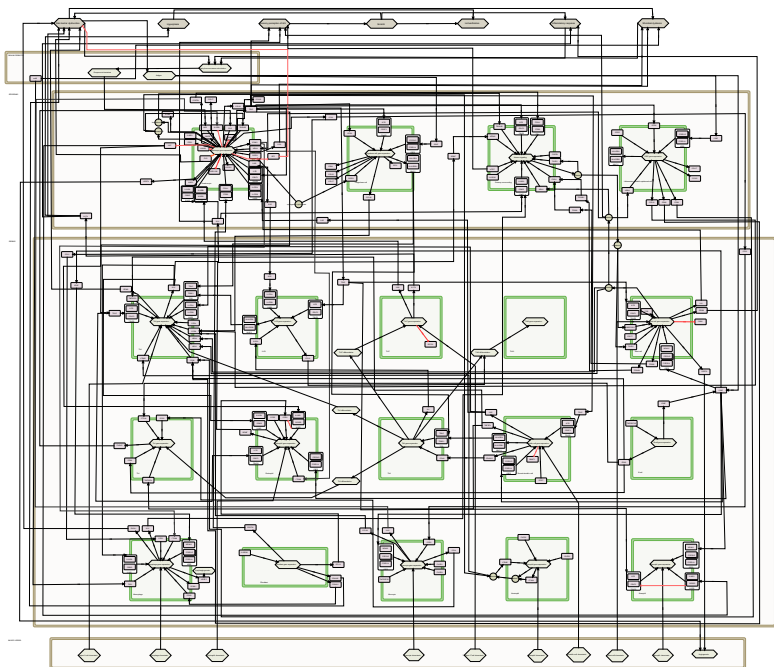

Supplement: Supplementary file 7 — Supplementary material [file mmc7.zip › AD_219.xml.pdf]

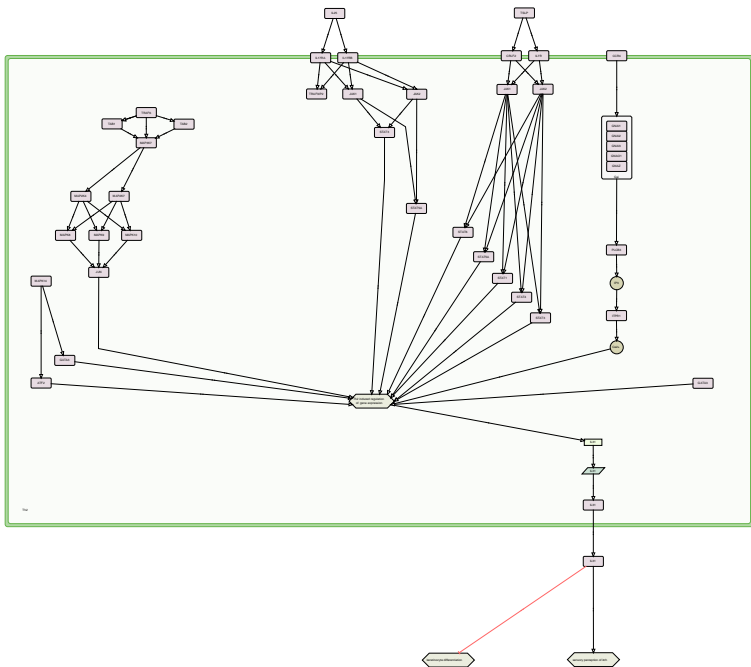

Supplement: Supplementary file 7 — Supplementary material [file mmc7.zip › AD_220.xml.pdf]

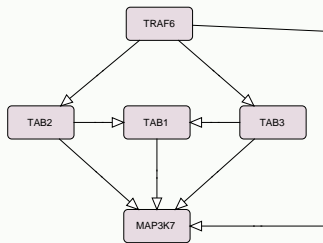

Supplement: Supplementary file 7 — Supplementary material [file mmc7.zip › AD_221.xml.pdf]

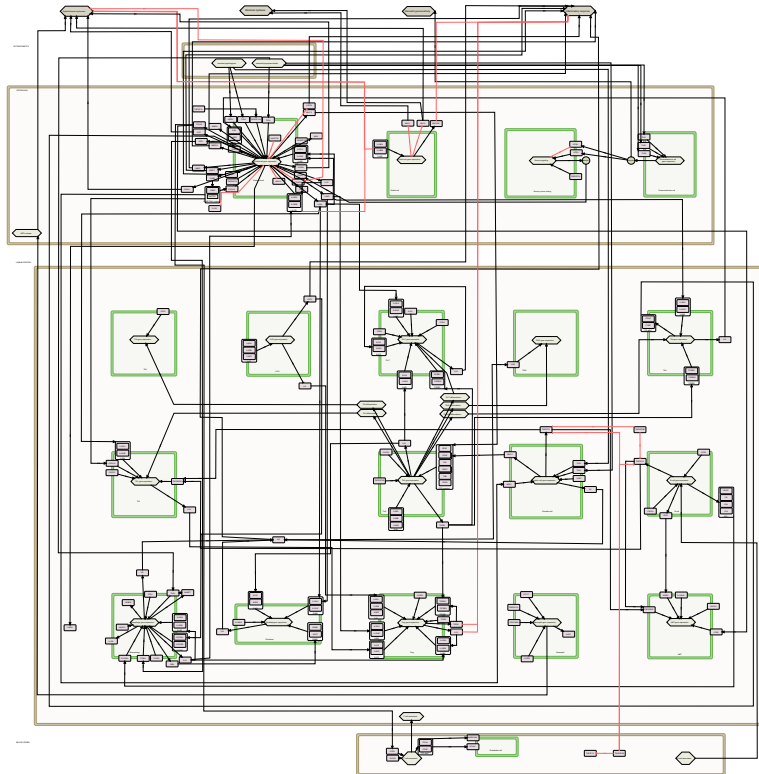

Supplement: Supplementary file 7 — Supplementary material [file mmc7.zip › UC_222.xml.pdf]

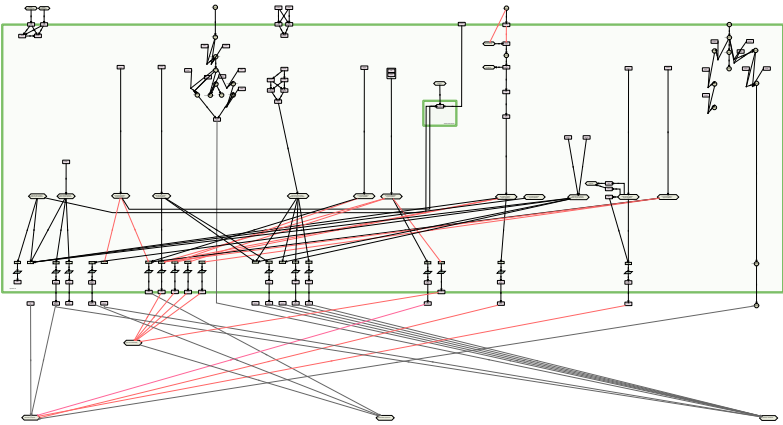

Supplement: Supplementary file 7 — Supplementary material [file mmc7.zip › UC_223.xml.pdf]
